# The evolution of transcription-associated biases of mutations across vertebrates

**DOI:** 10.1186/1471-2148-10-187

**Published:** 2010-06-18

**Authors:** Paz Polak, Robert Querfurth, Peter F Arndt

**Affiliations:** 1 Department of Computational Molecular Biology, Max Planck Institute for Molecular Genetics, Ihnestrasse 73, 14195 Berlin, Germany; 2 Department of Vertebrate Genomics, Max Planck Institute for Molecular Genetics, Ihnestrasse 73, 14195 Berlin, Germany

## Abstract

**Background:**

The interplay between transcription and mutational processes can lead to particular mutation patterns in transcribed regions of the genome. Transcription introduces several biases in mutational patterns; in particular it invokes strand specific mutations. In order to understand the forces that have shaped transcripts during evolution, one has to study mutation patterns associated with transcription across animals.

**Results:**

Using multiple alignments of related species we estimated the regional single-nucleotide substitution patterns along genes in four vertebrate taxa: primates, rodents, laurasiatheria and bony fishes. Our analysis is focused on intronic and intergenic regions and reveals differences in the patterns of substitution asymmetries between mammals and fishes. In mammals, the levels of asymmetries are stronger for genes starting within CpG islands than in genes lacking this property. In contrast to all other species analyzed, we found a mutational pressure in dog and stickleback, promoting an increase of GC-contents in the proximity to transcriptional start sites.

**Conclusions:**

We propose that the asymmetric patterns in transcribed regions are results of transcription associated mutagenic processes and transcription coupled repair, which both seem to evolve in a taxon related manner. We also discuss alternative mechanisms that can generate strand biases and involves error prone DNA polymerases and reverse transcription. A localized increase of the GC content near the transcription start site is a signature of biased gene conversion (BGC) that occurs during recombination and heteroduplex formation. Since dog and stickleback are known to be subject to rapid adaptations due to population bottlenecks and breeding, we further hypothesize that an increase in recombination rates near gene starts has been part of an adaptive process.

## Background

The double stranded structure of the DNA molecule and the Watson-Crick base pairing couples mutation processes on both strands. Every base X, on one strand pairs with just one complementary base X' on the other strand: G pairs with C and A with T. A mutation X→_F_Y on the forward strand is copied to a mutation X'→_R_Y' on the reverse strand. This is done either by repair enzymes that ensure the right base pairing of the DNA or during replication, if the mutation of one strand is not recognized by such enzymes. As a consequence, the rate of substitutions X→_F_Y of nucleotides X, Y on the forward strand, denoted by [X→Y]_F_, is equal to the rate of the complementary bases, X' and Y' on the reverse strand: [X→Y]_F _= [X'→Y']_R_. Under the assumption that mutations occur randomly on both strands and that the repair processes are equally effective on both strands we further have that [X→Y]_F _=[X→Y]_R _and therefore [X→Y]_F _=[X'→Y']_F_, i.e. that complementary substitutions occur with the same rate on one strand [1]. However this assumption and the symmetry of the substitution rates are not granted, since other cellular processes like transcription can distinguish the two strands. The signature of transcription associated substitution patterns is a particular substitution asymmetry in template and non-template strand [2,3] where the ratio [X→Y]/[X'→Y'] is different from one. Henceforth, rates will be calculated on the non-template strand, we therefore drop the subscripts F and R.

In bacteria and mammals, the process of transcription coupled repair (TCR) [4,5] can lead to strand asymmetries [6]. Additionally in Escherichia coli, cytosine deamination rates on the non-template strand are increased as a consequence of single strand DNA (ssDNA) formation of this strand during transcription [7-9]. In yeast, there is also a direct link between transcription and mutations occurring during recombination, which in turn is associated with transcription in this organism [10,11]. Recombination is positively correlated with higher fixation rates of strong nucleotides (S = G or C that form three hydrogen bonds with each other) over weak ones (W = A or T that form two hydrogen bounds with each other) [12]. Consequently the mutational signature of recombination is not a strand asymmetry, but rather an excess of substitutions of weak bases in strong bases over the reverse [13]. Similarly, cytosine methylation levels are positively correlated with CpG deamination rates [CpG→CpA/TpG] [14]. The association of transcription with repair, recombination and mutagenesis, has also been suggested in multi cellular organisms [2,15,16]. However, it has been technically very hard to study these associations at a genome wide level, since mutations occur at very low rates.

Comparative genomics between distantly related species is currently one of the strongest tools to study the impact of transcription on mutation patterns. The rate of substitutions, in particular genomic loci, is determined by regional mutagen levels, repair efficiency and selection. However, in regions that are presumably neutrally evolving, the estimated substitution rates are considered to be a good approximation of the actual mutation patterns. The increasing amount of sequenced genomes, and their corresponding alignments, allows estimating substitution rates in a large variety of species, with increasing resolution.

Previously, we investigated the substitution patterns along the non-template strand in 10 kbp regions centered on the transcriptional start site (TSS) and the 3'end of human genes [17]. Our analysis has revealed that five out of six possible symmetries between complementary single-base substitutions are broken in human intronic regions [17]. The next question is whether the substitution patterns associated with transcription in humans are different from those in non-human species? Since the average base composition of introns in different taxa are not uniform, the answer might be positive [18,19] and implies that mutational processes coupled to transcription vary across taxa.

In this work, we provide a comprehensive study of substitution patterns along genes and their flanking regions in ten vertebrate species of the four taxa: primates, rodents, laurasiatheria and bony fish. We have used a sliding window analysis to study the different substitution rates as a function of the distance from the 5'end and 3'end of genes. The inferred rates maximize the likelihood that all sites in a given multiple alignment are evolving according to a sequence evolution model, which is a continuous time Markov model. Our model provides lineage specific substitution rates, allowing us to address questions about the evolution of these rates. This model does not assume that the genomic base composition is at equilibrium (or stationary). Therefore, using the estimated lineage specific rates and under the assumption that the genomic regions will continue to evolve accordingly, we can estimate the future nucleotide compositions along genes in all lineages (which are not directly connected to the root of the tree). The resulting stationary base composition represents the expected content of As, Ts, Cs and Gs at equilibrium. According to this, in mammalian intronic regions, we found an excess of Ts and Gs over As and Cs in the stationary state. This deduced distribution is similar to the current nucleotide composition, suggesting that the current strand asymmetry have been generated by asymmetries in substitution rates.

Our analysis has revealed that strand asymmetries vary significantly between mammalian and fish genes. We also investigated the substitution patterns that impact the weak (W) and strong (S) base composition of the genomes. We found that the ratio of the substitution rates, [W→S] over [S→W] increases towards the 5'end of vertebrate genes. We show that this increase in [W→S]/[S→W] ratio is a consequence not only of a drop in CpG methylation-deamination rates [CpG→TpG/CpA] near the TSS, but also of another force that we speculate to be biased gene conversion. Furthermore, the stationary GC content (GC*) profiles peak near the TSS, although it does not exceed 50% in most species, while the current GC content is about 60%. Since a spike of GC content is a hallmark of TSSs in mammalian species, it is surprising that the mutational pressure implies a lower GC* than the current GC content in most mammals. However, in dogs GC* is higher than the current GC content, so regions surrounding the TSS become even richer in GC content. In sticklebacks, the average GC content frequency near the TSS is lower than 0.5, but the mutational forces imply GC* > 0.6. Hence, dog and stickleback are exceptions of expanding GC content near the TSS while in all other vertebrate species the GC content is decreasing.

## Results

We estimated substitution rates along genes and their 5 kb upstream and downstream flanking regions. In order to test for strand dependency of substitutions, we calculated the ratios between complementary transition rates along the non-template strand. In vertebrate genomes, the methylation levels are low in CpG islands [20], which lead to low methylation-deamination rates in these loci. Therefore, the analysis of mutational patterns was carried out separately for gene-promoters overlapping with CpG islands (CGI-genes) and for the rest of the genes (nonCGI-genes). As we previously reported, in general, strand asymmetries associated to transcription can be divided into global patterns that are detected along the whole transcribed region and local patterns where the asymmetry is restricted to the first 1-2 kbp of the transcript [17].

### Strand asymmetries are similar among mammals and stronger in CGI-genes

**Figure 1:**
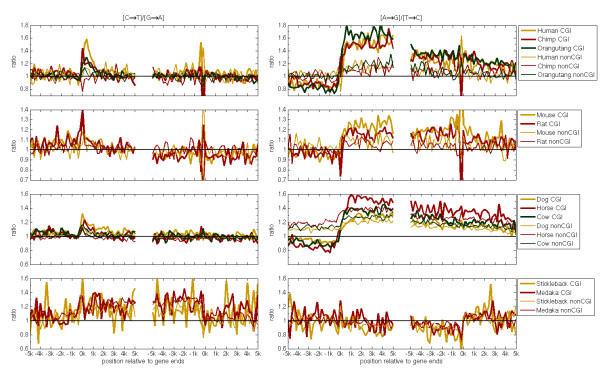
**Ratios of complementary transition frequencies across vertebrates**. There are four rows of panels and two columns of panels. The rows correspond to the four taxa analyzed in this study and the columns to the two types of ratios between complementary transition frequencies. Each panel consists of two sections: The left section is centered on the TSSs (left 0 k) and refers to 5 kbp of intergenic region upstream to the TSS and extends towards 5 kbp of intronic region downstream to the TSS. The right section on a panel is similarly centered on the 3'ends (right 0 k), while the analyzed regions extend from 5 kbp of intronic regions (upstream to the 3'end) to 5 kbp of intergenic regions downstream to the genes 3' ends. The ratios are plotted against the distance from the 5' and 3'ends of genes. Ratios are calculated along the non-template strand from pooled 200 bp windows of genes annotated for the reference species in each taxon. For CGI-genes the ratios are presented by thick lines, for nonCGI-genes ratios as thin lines.

Previously, we have found that the ratio [A→G]/[T→C] along the 5 kb region downstream to the TSS and 5 kbp upstream to the 3'end is larger than one and relatively constant along the analyzed transcribed regions [17]; we denote this pattern as a "global pattern" as it is detected along the whole transcript. In addition to this global pattern there is a localized pattern, where [C→T]/[G→A] is greater than one only along the first 1-2 kb of transcripts, while the ratio is close to one outside of this region [17]. Similarly, in this work we find the global [A→G]/[T→C] and the local [C→T]/[G→A] asymmetries in all mammals. Yet the global [A→G]/[T→C] asymmetry seems to decline towards the 3'ends of transcripts (Figure 1). And as observed previously in human, the "global" asymmetries extend into the intergenic regions downstream to genes [17]. Additionally, upstream to the TSS in CGI-genes, there is an opposite strand asymmetry where [A→G]/[T→C] < 1 [17], while in nonCGI-genes, the rates are either equal, as seen in primates and rodents, or greater than one as in laurasiatheria. Across mammals the global and local asymmetries are significantly more pronounced in CGI-genes than in nonCGI-genes. This implies that similar CGI-linked processes in mammals are causative for the more pronounced patterns in CGI-genes (Figure 1).

In addition to the breaking of the strand symmetry of transition rates, three out of four of the transversion rate pairs are not equal to each other (Additional file 1 and Additional file 2). In intronic regions of all mammalian species that were analyzed in this study, the ratios [G→T]/[C→A], [C→G]/[G→C] and [A→T]/[T→A] are greater than one. These three asymmetries begin at the 5'end of genes and extend beyond the 3'end of genes as we observed previously for the global pattern i.e. the profile of [A→G]/[T→C] (Figure 1). In mammalian upstream intergenic regions we have found that the direction of asymmetries switches at CGI-genes i.e. [C→G]/[G→C] and [A→G]/[T→C] are greater than one downstream to the TSS and lower than one upstream to the TSS (Additional file 2 and Figure 1). In contrast, [G→T]/[C→A] and [A→T]/[T→A] ratios are close to one in the 5' flanking regions of the genes in most mammalian species (Additional file 1 and Additional file 2).

### Strand asymmetries along fish genes are different from mammalian asymmetries

Strand asymmetries are also found in introns of non-mammalian species (Figure 1), but their directions are different in most cases. In stickleback and medaka introns, the [A→G]/[T→C] ratio is smaller than one, opposite to the asymmetry in mammals (Figure 1). And the ratio [C→T]/[G→A] is greater than one, as it is found in the first 1-2 kbp downstream the TSS of genes in mammals, but it is not restricted to the vicinity of the TSS in fish genes. In contrast to mammals, in fish there is no difference in the level of asymmetry between CGI-and nonCGI-genes.

### The current single nucleotide substitution rates lead to TA and GC skews

Over a long period of time, biases in substitution rates should accumulate and lead to skews in the base composition of complementary DNA strands [21]. Measurements along introns of the TA skew (=([T]-[A])/([T]+[A])) and the GC skew (=([G]-[C])/([G]+[C])) [19] have been shown to differ between species as well as in respect to their location relative to the TSS [18,19,22]. It is interesting to see whether the observed biases of intronic substitution rates can lead to similar skews as seen in current genomes. We have found that for all vertebrates this is indeed the case; the direction of stationary skews in intronic regions agrees with the current ones in CGI-genes (Additional file 3) and in nonCGI-genes (Additional file 4). Hence, the substitution rates can indeed build the current skews in the genome. For nonCGI-genes the degree of the stationary skews are similar to the current ones in mammals. However, in intronic regions of primate CGI-genes, the stationary TA and GC skews are greater than the current ones. This might be due to the simplicity of our genome evolution model, which does not include other mutational processes, such as insertion and deletions that might have been acting against the influence of substitutions on base composition around and within genes. Alternatively, it is possible that the mutational force that leads to skews has become stronger during primate evolution.

Another characteristic of both the TA and GC skews is spikes at boundaries of genes (Additional file 3 and Additional file 4). At the 5'end of genes there is a local increase in both skews. For example in primates and rodents the GC skew is above 0.1 while in the rest of the transcript the skew is below 0.06 (Additional file 3 and Additional file 4). However at the 3'ends the GC skew is negative in all species except for primates, where we do not see a GC skew. So the GC skew is in opposite directions at both ends of genes. TA skews are positive along genes and are stronger near gene boundaries in all species, while strongest at the 3'ends. The increase in skews in gene boundaries, in particular at the 5'ends, is found also in the stationary nucleotide composition in most of species computed solely from the nucleotide substitution rates (Additional file 3 and Additional file 4). This suggests that substitutional biases acting over evolutionary time scales have generated these spikes in the skews.

In the upstream regions of primate and laurasiatheria CGI-genes one might expect to find skews since in these regions there are strand asymmetries in substitution rates (Figure 1 and Additional file 2). However, in both taxa the TA and GC skews are close to zero in the 5' intergenic flanking regions of CGI-genes. This might imply that even though we observe that two out of six single-nucleotide substitution rates are strand asymmetric, it is not enough to induce non-zero skews. Indeed, in laurasiatheria, the stationary skews are absent, which indicates that other mutational processes compensate the impact of strand specific nucleotide mutations. But the negative stationary TA and GC skews in primates suggest that the strand asymmetries in substitution rates are able to induce strand asymmetries in nucleotide distribution in these regions (Additional file 3).

### Mutational pressure tends to increase the GC content in the vicinity of TSSs of dog and stickleback genes

**Figure 2:**
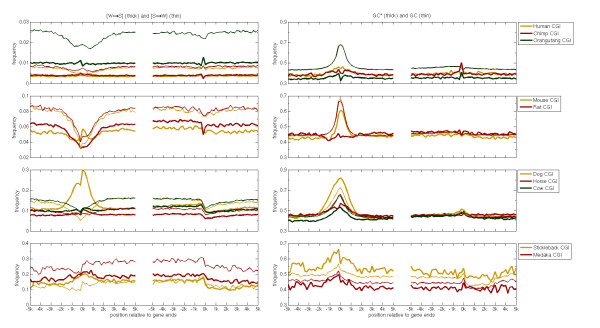
**The weak to strong bias along CGI-genes and their flanks**. The frequencies of W→S (thick line), S→W (thin line), the stationary GC content (GC*, thick) and the GC content (thin) are plotted against distance from the 5'end and 3'end of genes and calculated along the non-template strand from pooled 200 bp windows of genes annotated for the reference species in each taxon. Only the results for CGI-genes are presented.

Besides strand asymmetries we also investigated the impact of substitution rates on the GC content. All genomes of animals that have been sequenced so far are AT rich [23]. However, the GC content along the genome is heterogeneous [24,25], and the GC content in animals is higher near the TSS of genes than in other regions [26]. In particular, in mammals, the GC content near the TSS is higher by about 50% than on a genome wide level [26]. Several studies on the evolution of the GC content near the TSS debate whether selection has shaped the GC content or mutational mechanisms [13,19,26,27]. To address this, we used the estimated substitution rates in order to calculate the stationary GC content (GC*) along genes (Figure 2 and Additional file 5).

All mammals examined, except for the dog, show a clear peak in GC content at the TSS, while in the GC* profile the peak is lower indicating the loss of CpG islands. This trend is most pronounced in primate and rat CGI-genes, where the GC* profile is almost flat along the regions examined (Figure 2). This result is surprising, since CpG islands are usually believed to play a regulatory role in the transcription of genes [28] and therefore, one expects the current GC peaks to be preserved also in GC* profiles. Interestingly, given the close relation to human and chimpanzee, the orangutan genome shows a high rate of strong to weak substitutions leading to significantly lower GC content throughout the examined regions. In cow and horse genomes, GC* near the TSS is about 50%, which is higher than the value of 43% GC* in distal regions (Figure 2). Also at mouse TSSs, the expected GC* is, at 60%, well above 50% but still lower than the current GC content. However, most surprisingly, in dogs, the current GC content is about 60% at the TSS, while GC* is about 70%. So dog is the only mammalian specie where the recent nucleotide substitution rates lead to an increase in GC content around the TSS.

In stickleback, the GC* is higher than the current GC content along the regions we analyzed (Figure 2). The GC content at the 5'ends of CGI-genes is slightly greater than 50%. However, GC* is above 60% and hence stickleback promoters are expected to become richer in GCs than current mammalian promoters. Also in the intergenic regions, GC* is higher than in other species; it is around 50% at the upstream intergenic regions and about 48% in the regions downstream to the TSS (Figure 2).

### Methylation deamination rates drop near the TSS of vertebrate CGI-genes

One of the major mutational processes that impact the GC content along the genome is methylation of cytosines that are part of CpGs, which is by far the strongest mutational process in the mammalian genomes [14]. The rates of CpG to TpG or CpA substitutions are more than ten times higher than any other single base substitutions in the human lineage [29]. However, near the TSS of CGI-genes in vertebrates, we find that methylation-deamination rates drop close to zero (Additional file 6) as we have seen before in human genes [17]. In nonCGI-genes we also observe a decrease in the CpG methyaltion-deamination rates near the TSS but it is more moderate than in CGI-genes (Additional file 7). This difference in methylation-deamination rates at the proximity of TSSs between nonCGI-and CGI-genes is expected, since CGIs are subject to lower methylation levels compared to the rest of the genome [30] and consequently, CpGs in CGIs are less prone to deamination [20]. The impact of methylation-deamination can also increase the transversion rates in CpGs. Indeed, we observe that transversion rates of Cytosine or Guanine in CpG decline near TSSs of CGI-genes, but this decrease is more moderate than the one observed for transitions (Additional file 8 and Additional file 9). This implies that methylation related transversions and transitions are not caused by the same process.

### The ratio of [W→S]/[S→W] increases near the TSSs of vertebrate genes

GC* reflects the balance between the rates of W→S and S→W substitutions. Therefore, it is not surprising that the ratio [W→S]/[S→W] increases near the TSS of vertebrate genes. In particular this is true for CGI-genes in mammals and stickleback. In most species the change of cytosine into uracil occurs at higher rates than other single base mutations; therefore, at the genome wide level the ratio [W→S]/[S→W] is lower than one in animal genomes [3]. The rates of CpG methylation-deamination are significantly lower near mammalian TSSs than in the rest of the genome, which might induce the peak in the [W→S]/[S→W] profile near TSSs. However, even after excluding the CpG methylation-deamination rates in the calculation of the ratio [W→S]/[S→W] (using only the single-base substitution rates) we observe an increase of this ratio near the TSS of mammalian CGI-genes (Additional file 10). This ratio is greater than one in the vicinity of the TSS of laurasiatheria and mouse genes. In stickleback in particular, the ratio is greater than one along all analyzed regions. These results imply that in vertebrate non-CpG sites, the mutational pressure favors strong bases near the TSS.

## Discussion

### Mechanisms that violate the balance between [A→G] and [T→C] in transcribed regions

It has been proposed that the excess of [A→G] over [T→C] substitutions in mammals is a byproduct of transcription coupled repair (TCR). TCR is activated when RNA polymerase II stalls due to DNA lesions [5]. Green et al. [2] suggested that lesions that halt RNA pol II can be base mismatches that are formed during replication. There are two replication errors contributing to substitutions of A in G on the non-transcribed strand. The first error is misincorporation of Gs at template Ts when the non-transcribed strand is copied. The second error is misincorporation of Cs at template As during the replication of the transcribed strand. These errors result in G-T and A-C (non transcribed-transcribed) mismatches, which become the substitution-mutations A→G on the non-transcribed strand, when they are repaired into G-C base pairs. In a similar fashion, a substitution T in C on the non-transcribed strand is a result of misincorporations of Cs at template As and of Gs at template Ts, which results in C-A and T-G mismatches. When the base pair mismatches are repaired via the TCR pathway, the non-transcribed strand serves as a template for the correction of the transcribed strand. Therefore, the balance between the rates of [A→G] and [T→C] is determined by the balance between misincorporations and their repair. Since the miss-incorporation rate of purines into the strand being copied is higher than the rate of pyrimidines, the rate [A→G] is higher than of [T→C] on the non-transcribed strand.

The above model has been criticized by different groups mainly because mismatches in the DNA are not expected to halt transcription and therefore should not initiate TCR. An alternative model, that we suggest, is that mismatches are introduced by a removal of DNA lesions that block transcription via error-prone nucleotide-excision repair (NER) mechanisms. By this model, the DNA lesion is excised together with a surrounding DNA sequence and the resulting gap [31] is filled by a low fidelity DNA polymerase, such as DNA polymerase beta [32]. As a consequence the newly synthesized DNA is prone to accumulate mutations and to form DNA mismatches. Unfortunately, the in-vivo error spectra of DNA polymerases are not known and therefore without further studies we can not suggest DNA polymerases that might be the good candidates to explain the observed biases.

In a previous study we suggested that the mechanism, which generates the localized strand asymmetry near the TSS of human genes, is part of the somatic hyper mutation process (SHM see below); a processes that induces a genetic diversification of antibodies. In a recent paper, Steele reviewed the data on mutations, that result from SHM, along introns of genes that are part of the Immunoglublin locus [33]. Steele found that in this locus, on the non-transcribed strand, the mutation rates of A→G, A→T and A→C excess their reverse complement mutation rates. We also observe an excess of [A→G] over [T→C] and an excess of [A→T] over [T→A] along mammalian introns (Figure 1 and Additional file 2). But on the other hand, we find no or only a slight excess of [A→C] over [T→G] (Additional file 1). These similarities imply that somatic hyper mutation and TCR employ similar but not identical sets of proteins.

How are strand biases introduced in SHM? During somatic hyper mutation, the induction of the enzyme "Activation Induced Cytidine Deaminase" (AID) leads to the deamination of cytosines into uracils on the non-transcribed strand of immunoglobulin genes [34]. The U:G mismatches are assumed to invoke mutations in two phases [35]. At the first phase, the handling with U:G mismatches leads to transitions or transversions of C:G base pairs. If one strand is primarily targeted by AID, then the rates of C→T, C→G and C→A will exceed the rates of the reverse complement strand. At the second phase, mutations can occur in the surrounding sequence of the U:G mismatch mainly in A:T base pairs [33]. This has been suggested to be due to the removal of parts of the surrounding sequence containing an abasic site that is created when the uracil is excised by uracil-DNA glyocsylase (UNG). The resulting gap is then filled by the error prone DNA polymerase eta [36,37], promoting mutations of the type A→T. As a consequence, the non-transcribed strand of immunoglobulin genes accumulates transversions in A→T over T→A, as we also find in mammalian introns (Additional file 2).

Even though the before mentioned pathways can explain part of the global asymmetries along introns, there are two arguments against these hypotheses. First, in SHM the non-transcribed strand is often damaged and is also actively repaired [34], while in TCR, the transcribed strand is subject to repair processes [5]. Second, the bias of [A→G] over [T→C] can not be explained by the traditional models for SHM.

An intriguing model, which has been recently suggested for SHM, provides a mechanism that can explain the observed global asymmetries, including the excess of [A→G] over [T→C] on the non-transcribed strand, via a repair of the transcribed strand. Higher mutation rates in As on the non-transcribed strand of immunoglobulin genes have been postulated to be caused by repair of the transcribed strand, via a combination of reverse transcription and RNA editing [38]. By this model, a DNA strand that contains abasic sites and/or uracil is first transcribed into RNA. This RNA forms a secondary structure with hairpins. Adenosines in the RNA hairpins are known targets for the RNA editing enzyme adenosine deaminase (ADAR) and are converted to inosines [38]. In the next step, reverse transcription of such an edited RNA results in a DNA sequence that is inserted into the DNA strand instead of the damaged fragment. Compared to the original transcribed sequence the newly synthesized transcribed-strand accumulates several mutations T→C, T→A and T→G [38] that appear on the non-transcribed strand as A→G, A→T and A→C. By our results for introns, the substitution frequencies of A→G and A→T exceed their reverse complement substitution frequencies (Figure 1 and Additional file 2), while [A→C] is only slightly exceeding [T→G] (Additional file 1). This suggests that such a repair mechanism can introduce the global strand asymmetries along genes.

### Mechanisms that violate the balance between [A→G] and [T→C] in intergenic regions

The excess of [A→G] over [T→C] substitutions downstream to the 3'end might be a result of the non-template strand being signaled for TCR or due to the continuation of transcription downstream to the 3'end [39] or because of other processes that are not induced by transcription per se [40]. The opposite bias [A→G]/[T→C] < 1, which is found upstream to genes, might be indicative for antisense transcription [39] from CGIs [17]. An alternative explanation is that CGIs in mammals are origins of bidirectional replication and here the asymmetries are caused by different handling of the leading and lagging strand during replication [41]. Also we propose that in laurasiatherian nonCGI-genes, the fact that in the upstream regions [A→G]/[T→C] > 1 (Figure 1) might be due to poor annotation of genes in dog (where most of genes are projected from other species). Therefore, the TSS might be located further upstream in nonCGI-genes than predicted by computational algorithms.

### Mechanisms that break the [C→T] and [G→A] symmetry within genes

Again TCR can be causative for this asymmetry and as we previously pointed out; the localized asymmetry observed in mammals [17], could be caused by frequent single strand conformation of the non-template strand in the vicinity of the TSS. Since cytosine in ssDNA deaminates at higher rates than in double stranded DNA (dsDNA) [42], this can lead to the formation of the asymmetry in GC rich regions, in particular in CGIs, as we observe. Prolonged ssDNA conformation around the TSS can be caused by a high occupancy time of RNA pol-II or the formation of secondary structures like R-loops and G-loops, which are preferentially formed in GC-rich regions [39]. In fish, the excess of [C→T]/[G→A] over one is not restricted to the first 2 kb as it persists along the whole transcript (Figure 1). Such an excess is found also in bacterial transcribed regions due to ssDNA conformation during transcription [9]. Hence, we suggest that the non-template strand of fish genes, which are shorter than mammalian genes, is found at ssDNA conformation at higher frequencies than in mammals. We predict that mammals might have developed mechanisms that prevent ssDNA formation outside of the first 2 kbp of transcripts. Lastly, the lower ratios of both [C→T]/[G→A] and [A→G]/[T→C] within intronic regions of nonCGI-genes can reflect a lower expression level of these genes during early development and in germ line cells [20,30], which would lead to a lower activity of transcription and TCR.

### Mechanisms that increase the rates of W→S over S→W

A molecular process that has been suggested to increase the rates of W→S over S→W substitution is the GC-biased gene conversion (BGC), which arises as a consequence of the GC-biased repair of G/C:A/T mismatches, that are formed in heteroduplex intermediates during recombination events in meiosis [13]. The BGC process, is not a mutagenic process that introduces *de-novo *mutations in the genome, but instead it increases the fixation probability of GC alleles over AT alleles [43,44]. Therefore BGC is assumed to act as a positive selective force for GC alleles with a selective coefficient that correlates with the recombination rates. In contrast, for S→W mutations, BGC acts as purifying selection force. Hence, BGC would increase the rate of W→S substitutions while decreasing the rate of S→W substitutions. This pattern is similar to the one that we observe near TSSs of CGI-genes in dog, cow, horse, stickleback and medaka (Figure 2). In rodents, the picture is slightly different. In contrast to other mammals, at the 2 kb regions centered at the TSS, the substitution frequencies are lower than in regions that are found at a distance of 1-5 kbp from the 5'ends. However, the drop in rates implies that negative selection near TSSs is stronger for mouse genes than in the rest of mammalian species. Within the 2 kbp long regions centered in mouse CGI-gene TSSs, there is an increase (and a decrease) in [W→S] ([S→W]) (Figure 2). In sum, the signature of BGC mutations rates is found near the TSS in all mammals and fish. However, these substitution patterns can also be formed due to a positive selection on the local GC content near the TSS. A pair of substitutions, which does not impact the GC content but has been found to be positively correlated with recombination rates is G:C→C:G [12]. In dog genes, these rates also increase in the vicinity of the 5'end of genes, in particular in CGI-genes. Therefore, we suggest that the BGC rates have shaped the patterns of [W→S]/[S→W] ratios in the vicinity of the TSS in all species.

### Chromosome length correlates with [W→S]/[S→W]

Previous studies have revealed that [W→S]/[S→W] is significantly higher in dogs than in any other mammal. It has been speculated that this is due to the shorter length of dog chromosomes, which is suggestive for higher recombination rates along these chromosome [3]. A support for this theory is that chicken micro-chromosomes, compared to macro-chromosomes, have a higher GC content, a higher observed/expected ratio of CpG, and a higher density of CpG islands [45]. This observation was recently extended to vertebrate genomes, in which the above three genomic features are anticorrelated with the chromosomal length [46]. Therefore, two species that have a similar population size but different average chromosomal length would have different [W→S]/[S→W] ratios. Dog, which has the shortest chromosomes among the mammals studied here, is expected to have the highest recombination rates.

In a similar fashion, one can explain the difference between stickleback and medaka. The average medaka chromosome is about 29 Mb long, 1.3 times longer than that of stickleback (22 Mb). If recombination rates are roughly reversely proportional to the chromosome length in stickleback, then the recombination rates should be about 1.3 times higher than in medaka. The value of [W→S]/[S→W] in non-CpG sites in stickleback is about 1.3 compared to the ratio of 0.85 in medaka, that is, 50% greater. This difference can also explain the higher density of CGIs in the stickleback (150 CGI/Mb) genome compared to the medaka genome (37 CGI/Mb) [46].

### Recombination rates are higher near the TSS due to adaptation

Compared to cow and horse, in dog genes the ratio of [W→S] over [S→W] is only higher near the TSS. Higher recombination rates along dog chromosomes can not solely explain the peak in [W→S]/[S→W] near the TSS. Interestingly, dog and stickleback, which have the highest [W→S]/[S→W] ratios, are the species that have experienced the greatest number of adaptation events or bottlenecks among the studied species [47,48]. The evolution of regulatory elements is often considered to be the prime driver of adaptation. Hence, it is possible that recombination rates are higher at the TSS in both dog and stickleback genes, due to the selection of newly recombinant promoter regions. The recombination rates along the genome are heterogeneous and determined by unknown factors; the rates themselves have been constantly changing during evolution. The recombination maps in human and chimpanzee reveal low number of overlapping hotspots [49,50]; the reason for that is not clear, but it is possible that selection can shape these rates across the genome during evolution. For example, in species like stickleback, which constantly adapt to new environments, recombination rates are expected to be higher near the TSS, since crossing over events can increase the fitness of the specie in a new environment. Another scenario is that recombination rates do not differ significantly between species, but the fixation rate of W→S substitutions increases near the TSS due to positive selection on GC rich motifs [26]. However, the fixation dynamic of these GC-alleles is similar to the one that is caused by BGC [44] and therefore these scenarios are indistinguishable by sequence comparison [43].

### Insights on evolution of transcription patterns

So far we have tried to explain the substitution patterns using biases in molecular processes, but there is the possibility that substitution profiles have been shaped by selection. For example, the strand asymmetries can be due to DNA motifs like transcription factor binding sites or splicing enhancers. Such motifs can be strand specific and their distribution can generate the observed strand biases [51]. In the near future, the increasing amount of experimentally verified regulatory elements will allow quantifying the contribution of selection to the strand asymmetries in substitution rates.

### Insights on the evolution of transcriptional processes across vertebrates

The substitution patterns in genes and their flanks are an evolutionary foot print of transcriptional processes. The fact that substitution asymmetries in CGI-genes are stronger than in nonCGI-genes could be indicative for CGI-genes being transcribed more often across mammalian germline cells. In addition, the inverse direction of the asymmetries [A→G] and [T→C] in upstream-intergenic regions compared to downstream-intronic regions of CGI-genes suggests that CGI-genes are origins of bi-directional transcription in mammals. On the other hand, the excess of [C→T] over [G→A] in intronic regions of CGI-genes, but not in the upstream regions, implies that there is a preference for one direction over the other. This suggests that substitution patterns can be used as a telescope to watch on the past of transcriptional processes.

## Conclusions

In this study we have found that within intronic regions, strand asymmetries between complementary substitution rates are found in all mammals. The profiles of the strand asymmetries are similar among mammals. We have found that strand asymmetries might be distinct in two different classes of genes. The excess of [C→T] over [G→A] is found in the 2 kb long regions downstream to the TSS of CGI-genes but not in non-CGI genes. Other strand asymmetries were found in both classes of genes but the degree of the asymmetries was reduced in non-CGI genes. This might imply that TCR is more active in CGI-genes, maybe due to their higher expression in germ cells.

In fish genomes we find other patterns of asymmetries than in mammals. The excess of [A→G] over [T→C] that is found along mammalian genes is not present in fish genes, where we observe an opposite bias. Another difference is the fact the localized asymmetries are absent in fishes. We hypothesize that this difference is related to differences in TCR and mutagenic processes between fish and mammals.

Another topic that has been addressed in this work is the bias between [W→S] and [S→W]. The ratio [W→S]/[S→W] peaks at the TSS in most genomes. We suggest that this profile has been generated by BGC. The substitution rates also lead to a peak in the GC* profile near the TSS, but it can not explain the high current GC content near the TSS, since in most species GC* is lower than the current GC content. Moreover, in the majority of species, GC* is only slightly higher at the TSS than in further distal regions. The two exceptions are dog and stickleback, where GC* at the TSS is higher than the current GC content. We hypothesize that in these two species recombination in the promoters occurs or has occurred at higher rates than in other species and similarly biased gene conversion. Causative for high recombination rates might be the fact that both species have undergone strong selection through adaptation or breeding.

## Methods

### Substitution analysis

We estimated the substitution frequencies for all genes with respect to the non-template strand (i.e. the not transcribed or coding strand). We estimated the profiles of twelve single nucleotide substitution frequencies [X→Y]. In addition, due to the impact of methylation on mutation frequencies in vertebrate cells, six context dependent substitution processes of CpGs into TpG, CpA, ApG, CpT, CpC and GpG, have been taken into account and their frequencies quantified. Substitution frequencies have been estimated from multiple alignments of genomic sequences (see below). We used a maximum likelihood approach which correctly handles effects due to back-mutations [12] and is able to reliably estimate substitution frequencies from given aligned sequences along all branches that are not directly connected to the root node (the node that represents the last common ancestor of all species in a given tree) [17]. In order to estimate frequencies we assume that sequence evolution is a time irreversible Markovian process. In contrast to many models, we assume that the molecular clock might differ between different branches. Therefore, we estimate the 18 substitution frequencies along each branch of the phylogeny. Another difference between our model and other commonly used sequence evolution models is that we do not assume that the DNA nucleotide composition is at equilibrium. These relaxations make our model very general in comparison with other models.

We measure substitution frequencies per base pair, estimating the (fractional) number of nucleotide exchanges from one nucleotide to another along each branch of the phylogeny. We may compute the corresponding substitution rates (measured per bp and time) by dividing frequencies in the time that passed along a branch. However, in this study we are interested in the spectra of substitutions i.e. the relative difference between two substitution processes and therefore work with the frequencies only.

### Sequence annotation and multiple alignments

We analyzed the substitution frequencies of ten species: Human (Homo sapiens), Chimpanzee (Pan troglodytes), Orangutan (Pongo pygmaeus), Mouse (Mus musculus), Rat (Rattus norvegicus), Dog (Canis familiaris), Cow (Bos Taurus), Horse (Equus caballus), Stickleback (Gasterosteus aculeatus) and Medaka (Oryzias latipes). For the purpose of our analysis; mammals were grouped into the three clades of primates, rodents and laurasitheria (including dog, cow and horse). For mammals, gene annotation and multiple species alignments were downloaded from ensemble v55 [52]. For primates we used 4-way catarrhini-specific EPO alignments [53], for rodents and laurasiatheria the twelve amniota vertebrates EPO alignments [53]. The Enredo and Pecan (which together with Ortheus) comprise the EPO pipe line assures the consistency of the alignments with paralogs [53]. For fish gene annotation and Multiz 3-way alignments were downloaded from UCSC [54]. In each clade analyzed, gene annotation of just one species was used to determine the regions of interest. That is in primates: human (Ensembl v55), rodents: mouse (Ensembl v55), laurasitheria: dog (Ensembl v55) and in fish: stickleback (Ensembl v55). CGI coordinates for human, mouse, dog and stickleback were taken from the Ensembl (v55), and CpG island defined by: minimum length 400 bps; minimum GC content is 50%; and minimum ratio of observed CpG over expected is 0.6. In our analysis we used only protein coding genes and divided them into two classes: (1) CGI-genes, where the 5'end is found within a CpG island; (2) And nonCGI-genes, where 5'end is not located in a CpG island (see Additional file 11). The number and proportion of CGI-genes and nonCGI-genes vary among the references species. In human we used 9021 CGI-genes and 4786 nonCGI-genes and in mouse 6448 and 6708, respectively. Among the dog genes 2882 were CGI-related and 9262 nonCGI-related. The frequencies were then estimated from multiple alignments and the following phylogenies. Primates (((human, chimpanzee), orangutan), rhesus), rodents: ((mouse, rat), human), laurasiatheria (((dog, horse), cow), human) and ((stickleback, medaka), tetraodon).

### Dataset construction

The main goal of our analysis is to investigate the dependence of nucleotide substitution frequencies in transcribed regions and their 3' and 5' flanks. To do so, we estimate 18 substitution frequencies within 10 kb regions equally surrounding the transcript starts and similarly around the ends of transcripts (see [17]). We estimate the frequencies in the four taxa (primates, rodents, laurasiatheria and fish) independently by using a sliding window analysis at a resolution of 200 bp. An estimation of 18 substitution frequencies for DNA sequences of a few hundred bp in length will be dominated by stochastic noise. Therefore, in order to estimate the frequencies at specific positions relative to the gene ends, we used genome-wide pooled 200 bp long non-overlapping windows, which are located at fixed distances from individual 5'ends (or 3'ends) while masking all annotated exons (Additional file 11). For each gene we defined intronic and intergenic flanking regions around its 5' and 3'ends (Additional file 11). The ends of genes are given by Ensembl annotations and they are defined as the furthest 5' and 3'ends among all transcripts of one gene (alternative TSSs were ignored). The boundaries of the analyzed regions were the middle points between the gene and its neighbor genes. Since we restrict the analyzed regions to a distance of 5 kbp from either 5' or 3'ends of genes, the maximal distance of a window from one of the genes ends is 5 kbp (Additional file 11). But since we include genes shorter than 5 kbp, in these cases the analyzed regions will be truncated and, hence, shorter than 5 kbp (Additional file 11). For example, genes of length 3 kbps will not intersect with windows that are found at a distance of 3 to 5 kbp downstream to the TSS. Hence, this gene will not contribute to the pool of intronic sequences at these distances. The same is true for genes with either upstream to the 5'ends (or downstream to 3'ends) intergenic regions shorter than 10 kbp (Additional file 11). Similarly, intronic windows that overlap (partly or completely) with exons residing on the opposite strand are truncated to exclude all exonic regions (Additional file 11). The substitution frequencies have been estimated from pooled alignments of genomic sequences of up to 4 representative species in each taxon (see above).

### Estimation of [W→S] and [S→W] frequencies

The frequencies of substitutions of weak base (W = A or T) to strong base (S = C or G) and *vice versa *are calculated as follows:

Where [X→Y] is the frequency of substitutions of base × in Y. The density of base × in a bin is denoted by [X]; [CpG] is the density of the CpG di-nucleotides.

## Competing interests

The authors declare that they have no competing interests.

## Authors' contributions

PP conceptualized and conducted the study, analyzed the data and wrote the initial draft of the manuscript. PFA and RQ participated in conceptualization, discussion, writing and editing of the manuscript. PFA supervised and coordinated the work. All authors read and approved the final manuscript.

## Supplementary Material

Additional file 1 **Profiles of [G→T]/[C→A] and [T→G]/[A→C] ratios across vertebrates**. The ratios are plotted against distance from the 5' and 3'ends of genes and are calculated along the non-template strand from pooled 200 bp windows of genes annotated for the reference species in each clade. For CGI-genes the ratios are presented by thicker lines. Click here for file

Additional file 2 **Profiles of [C→G]/[G→C] and [A→T]/[T→A] ratios across vertebrates**. The ratios are plotted against distance from the 5' and 3'ends of genes and are calculated along the non-template strand from pooled 200 bp windows of genes annotated for the reference species in each clade. For CGI-genes the ratios are presented by thicker lines. Click here for file

Additional file 3 Current and stationary TA(*) and GC(*) skews along CGI-genes and their flanks. Current stationary skews are plotted with thicker lines. The skews are plotted against distance from the 5' and 3'ends of genes and are calculated along the non-template strand from pooled 200 bp windows of genes annotated for the reference species in each clade. Click here for file

Additional file 4 Current and stationary TA(*) and GC(*) skews along nonCGI-genes and their flanks. Current stationary skews are plotted with thicker lines. The skews are plotted against distance from the 5' and 3'ends of genes and are calculated along the non-template strand from pooled 200 bp windows of genes annotated for the reference species in each clade. Click here for file

Additional file 5 **The weak to strong bias along nonCGI-genes and their flanks**. The frequencies of W→S (thick lines) to S→W (thin lines), the stationary GC content (GC*, thick) and the GC content (thin) are plotted against distance from the 5'end and 3'end of genes and calculated along the non-template strand from pooled 200 bp windows of genes annotated for the reference species in each clade. Click here for file

Additional file 6 **The frequencies of W→S, S→W and CpG methylation deamination process around the ends of CGI-genes in vertebrates**. The frequencies S→W (thin lines) are calculated without the substitution in CpGs and compared to W→S frequencies (thick lines). The methylation deamination rates CpG→TpG (thick) and CpG→CpA (thin) are presented in the right panels. See Additional file 1 for further details. Click here for file

Additional file 7 **Portable Network Graphics (PNG)**. The frequencies of W→S, S→W and CpG methylation deamination process around the ends of nonCGI-genes in vertebrates. The frequencies S→W (thin lines) are calculated without the substitution in CpGs and compared to W→S frequencies (thick lines). The methylation deamination rates CpG→TpG (thick) and CpG→CpA (thin) are presented in the right panels. See Additional file 1 for further details. Click here for file

Additional file 8 **Transversion rates that are associated with CpG methylation deamination process around the ends of CGI-genes in vertebrates**. The methylation deamination rates CpG→CpT (thick) and CpG→ApG (thin) are presented in the left panels. The rates CpG→GpG (thick) and CpG→CpC (thin) are presented in the right panels. See Additional file 1 for further details. Click here for file

Additional file 9 **Transversion rates that are associated with CpG methylation deamination process around the ends of nonCGI-genes in vertebrates**. The methylation deamination rates CpG→CpT (thick) and CpG→ApG (thin) are presented in the left panels. The rates CpG→GpG (thick) and CpG→CpC (thin) are presented in the right panels. See Additional file 1 for further details. Click here for file

Additional file 10 Profiles of [W→S]/[S→W] and GC*/GC ratios across vertebrates. For CGI-genes the ratios are presented by thicker lines. See Additional file 1 for further details. Click here for file

Additional file 11 **Sketch of the analyzed regions around the 5' ends of two classes of genes**. Genes starting within CGIs, denoted by striped boxes, and genes without CGI. CGIs are denoted by striped boxes. The template strand for transcription (also denoted by the coding strand) is the reference strand that is used for the substitution analysis and for defining the directionality 5'→3' relative to the 5' end (which denoted by 0) gene start (broken arrow). The substitution analysis was done in the 10000 bp long regions centered on the 5'end of gene (denoted by the two outmost vertical lines). This region of analysis was further truncated if the next upstream gene was closer than 10000 bp (white box) or the 3'end of the gene was closer than 5000 bp. Further all, exons were excluded (gray boxes). Bold black lines depict the finally analyzed sequences. The substitution frequencies are estimated relative to the 5'end of genes using a sliding window analysis. Click here for file
