# Supplementary figures and images for: The evolution of transcription-associated biases of mutations across vertebrates
Source: BMC Evol Biol. 2010 Jun 18;10:187. doi: 10.1186/1471-2148-10-187 (PMC2927911; doi:10.1186/1471-2148-10-187)

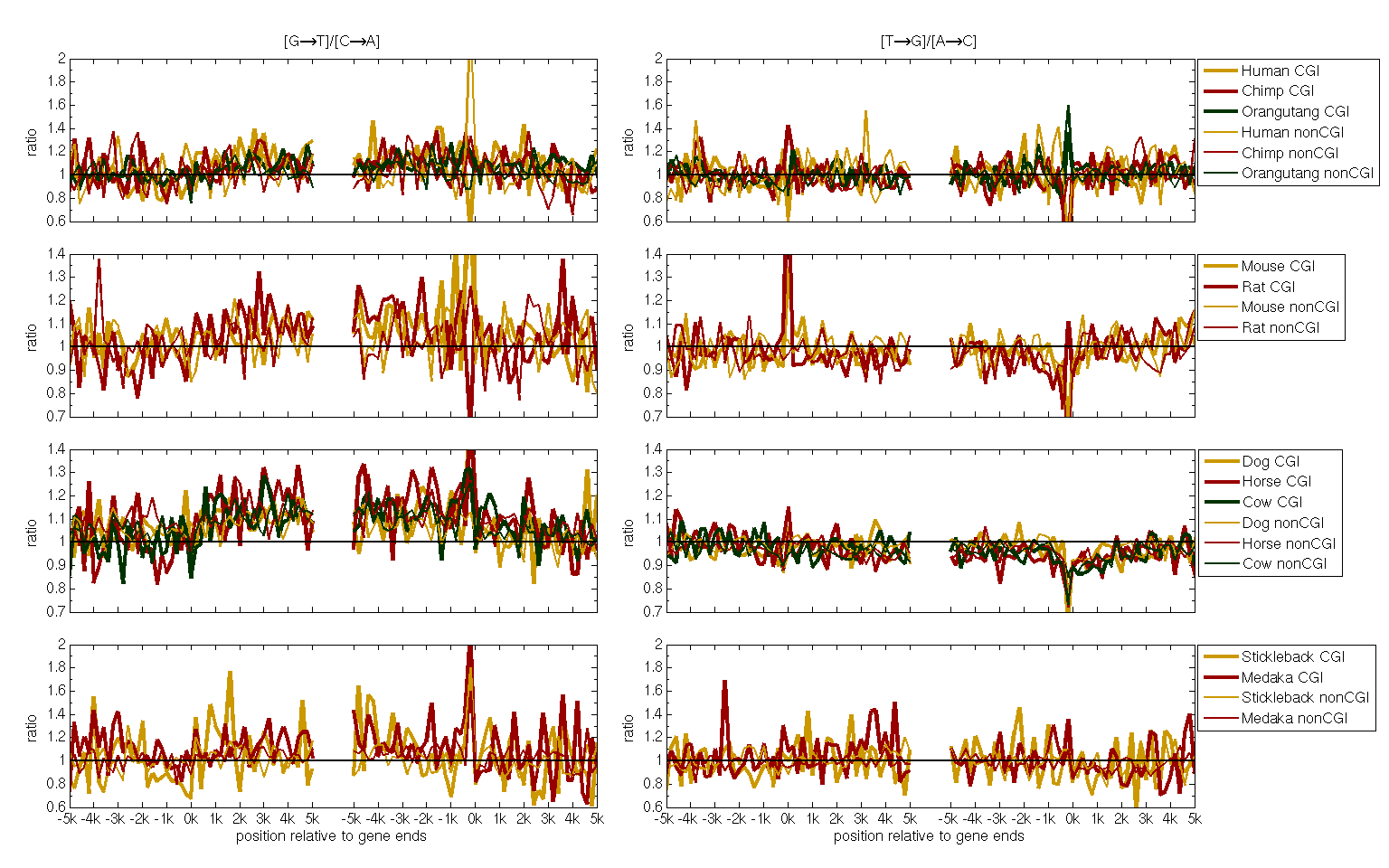

Supplement: Additional file 1 — Profiles of [G→T]/[C→A] and [T→G]/[A→C] ratios across vertebrates. The ratios are plotted against distance from the 5' and 3'ends of genes and are calculated along the non-template strand from pooled 200 bp windows of genes annotated for the reference species in each clade. For CGI-genes the ratios are presented by thicker lines. [file 1471-2148-10-187-S1.PNG]

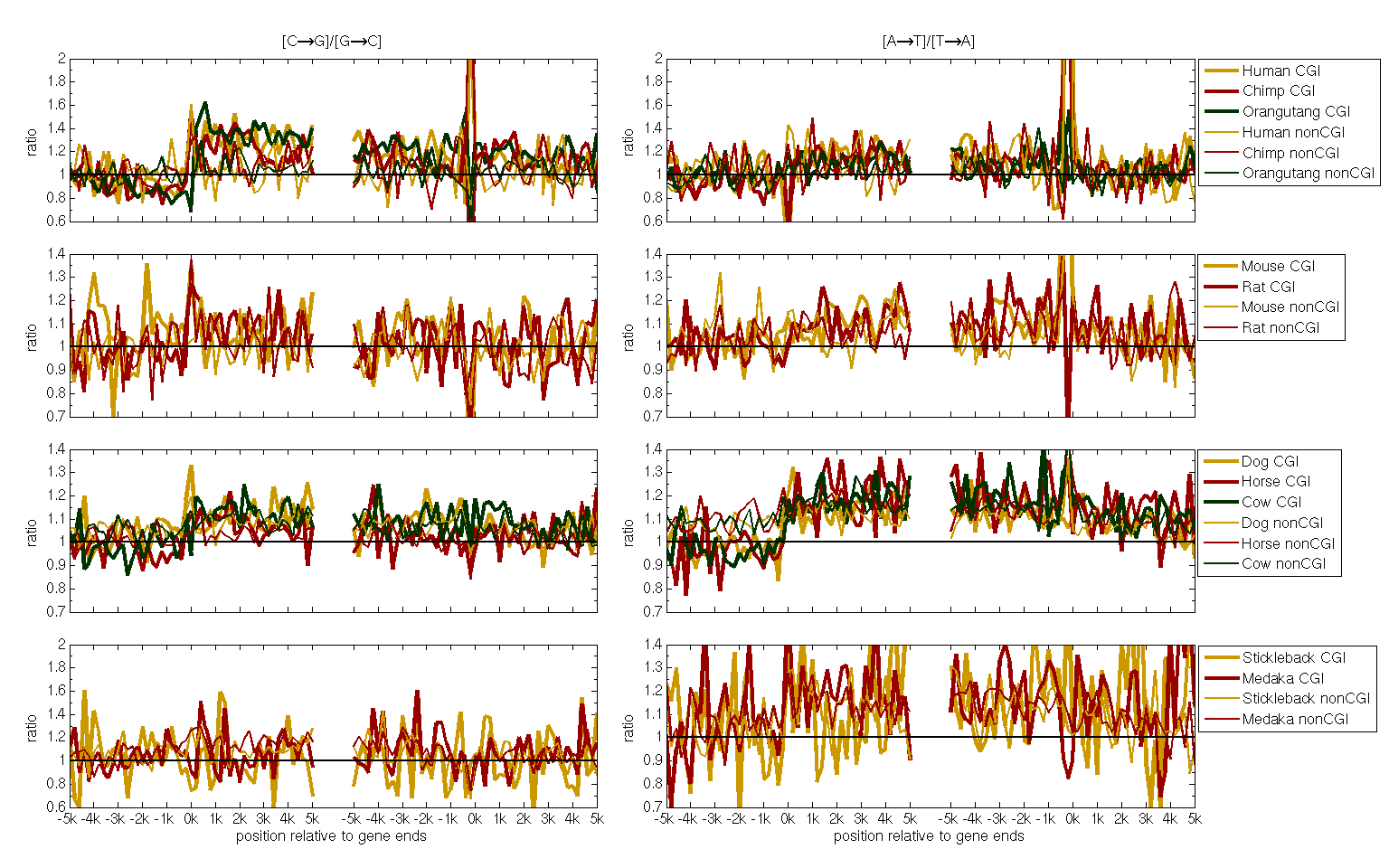

Supplement: Additional file 2 — Profiles of [C→G]/[G→C] and [A→T]/[T→A] ratios across vertebrates. The ratios are plotted against distance from the 5' and 3'ends of genes and are calculated along the non-template strand from pooled 200 bp windows of genes annotated for the reference species in each clade. For CGI-genes the ratios are presented by thicker lines. [file 1471-2148-10-187-S2.PNG]

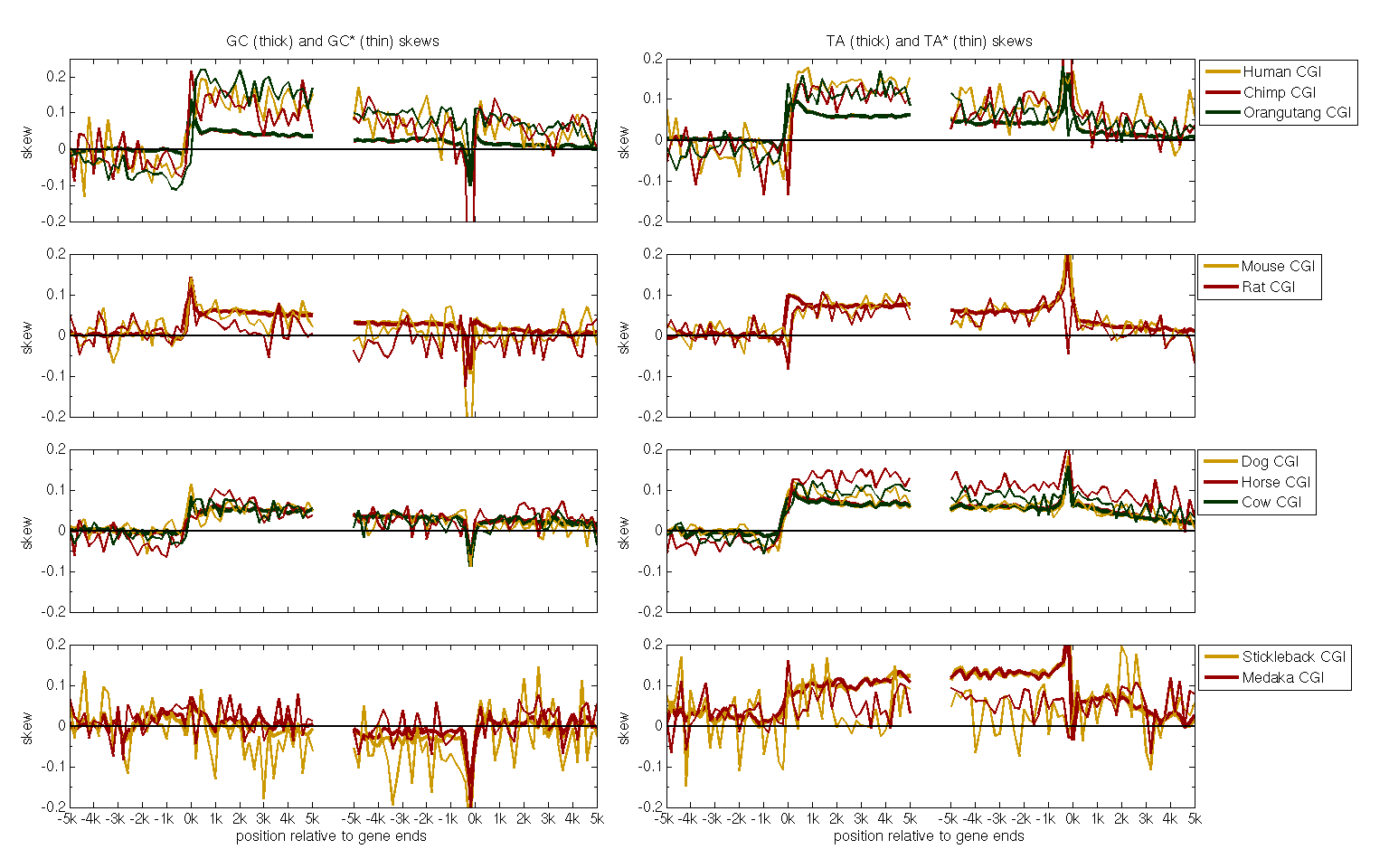

Supplement: Additional file 3 — Current and stationary TA(*) and GC(*) skews along CGI-genes and their flanks. Current stationary skews are plotted with thicker lines. The skews are plotted against distance from the 5' and 3'ends of genes and are calculated along the non-template strand from pooled 200 bp windows of genes annotated for the reference species in each clade. [file 1471-2148-10-187-S3.PNG]

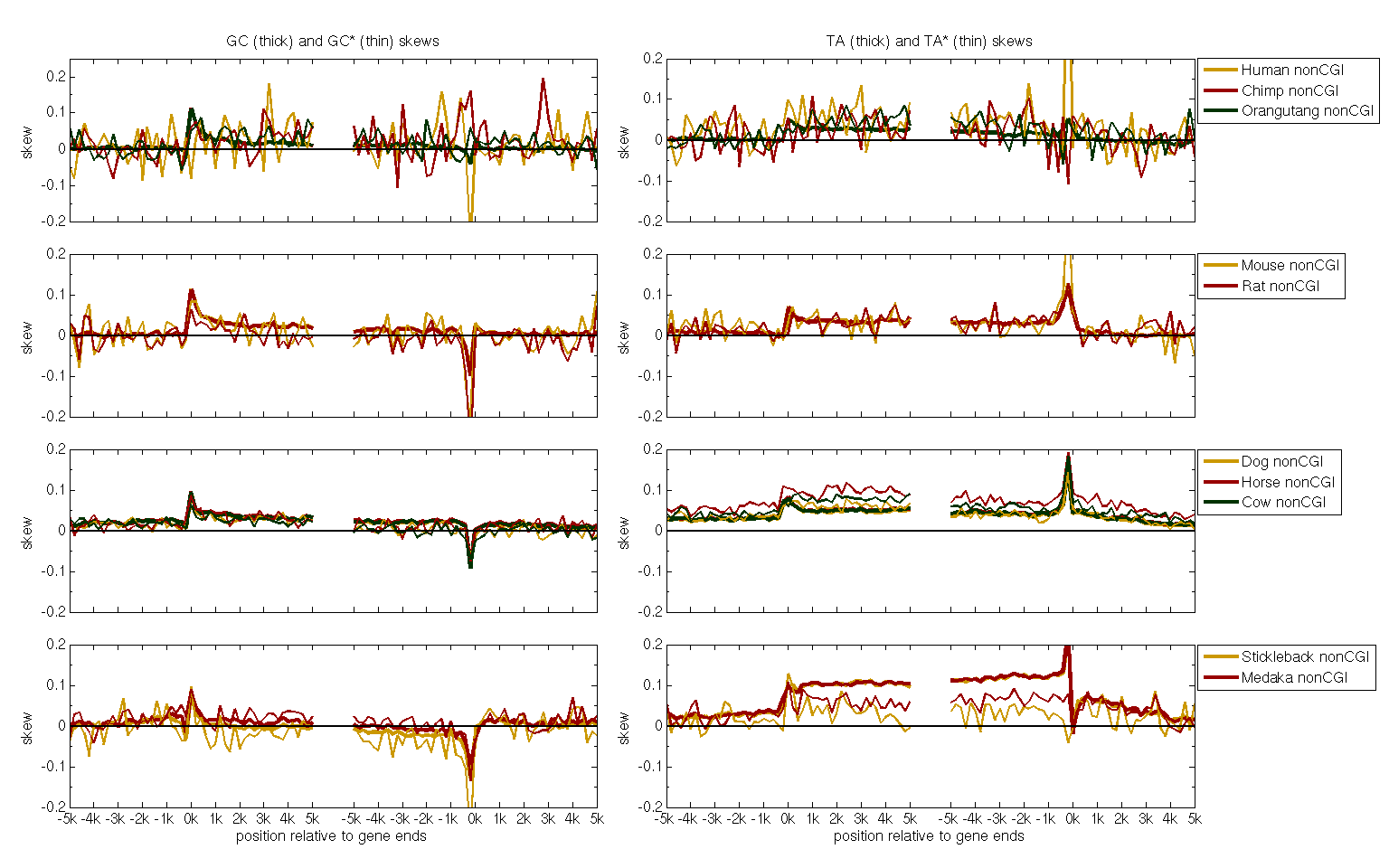

Supplement: Additional file 4 — Current and stationary TA(*) and GC(*) skews along nonCGI-genes and their flanks. Current stationary skews are plotted with thicker lines. The skews are plotted against distance from the 5' and 3'ends of genes and are calculated along the non-template strand from pooled 200 bp windows of genes annotated for the reference species in each clade. [file 1471-2148-10-187-S4.PNG]

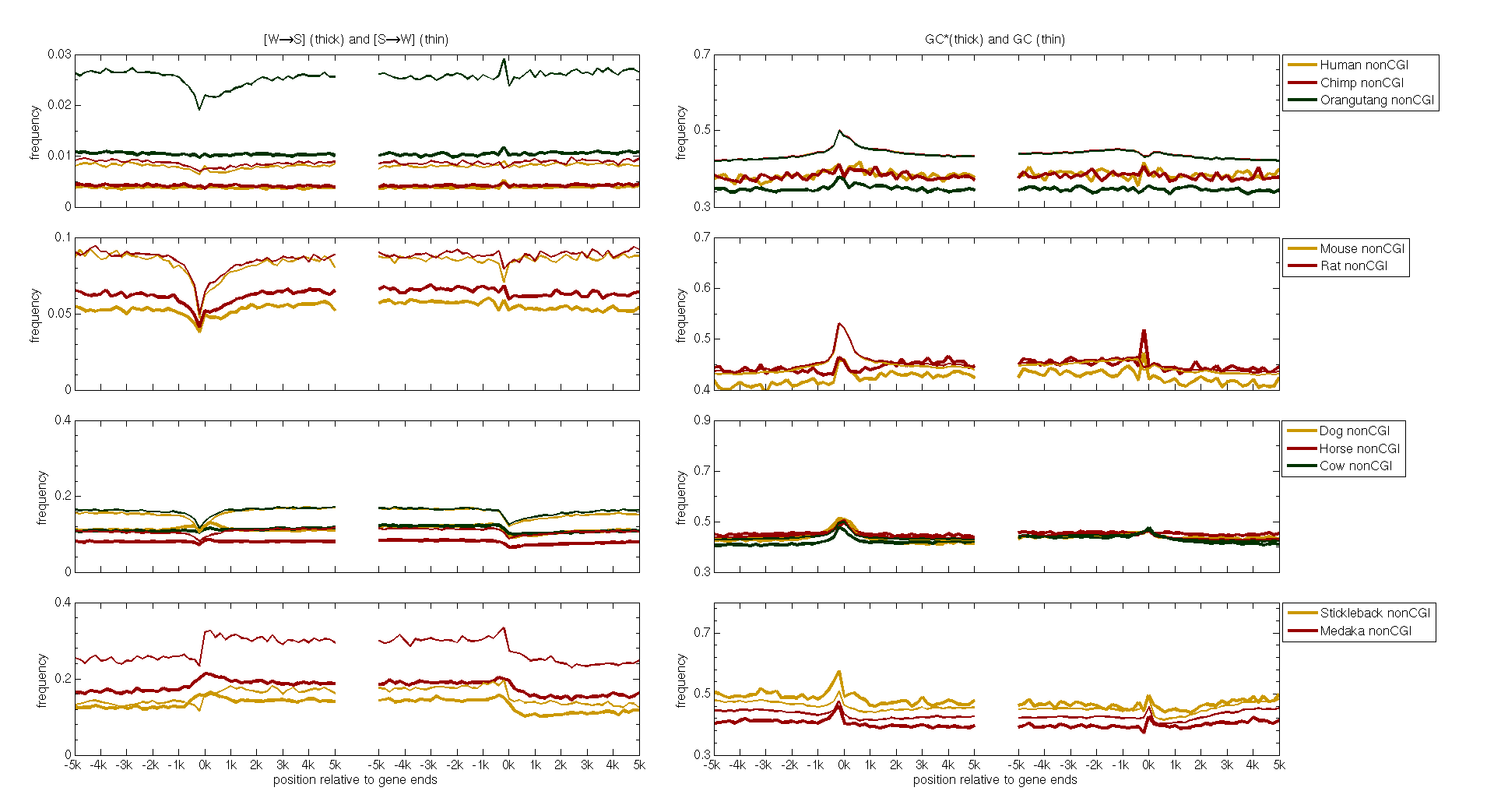

Supplement: Additional file 5 — The weak to strong bias along nonCGI-genes and their flanks. The frequencies of W→S (thick lines) to S→W (thin lines), the stationary GC content (GC*, thick) and the GC content (thin) are plotted against distance from the 5'end and 3'end of genes and calculated along the non-template strand from pooled 200 bp windows of genes annotated for the reference species in each clade. [file 1471-2148-10-187-S5.PNG]

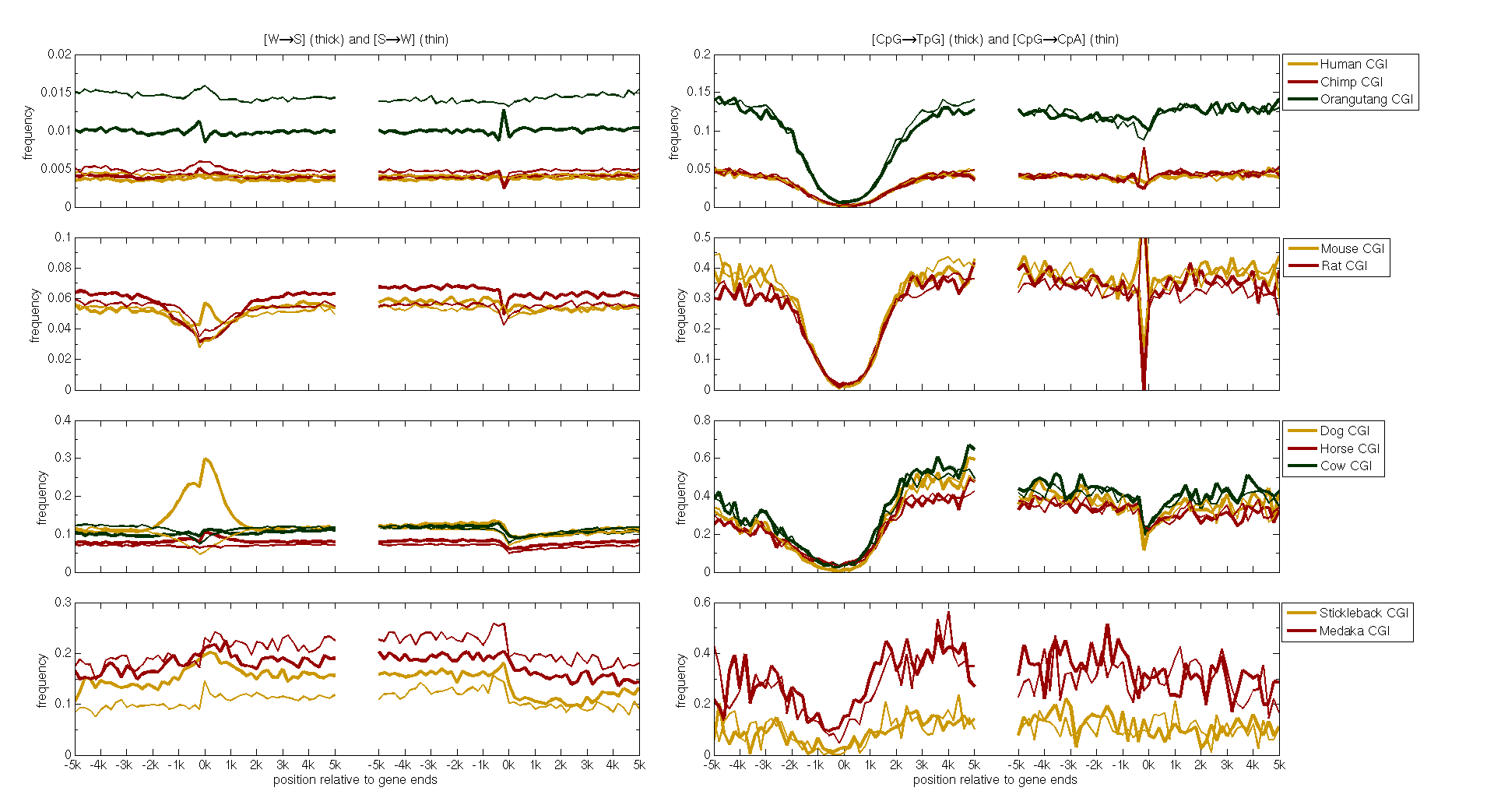

Supplement: Additional file 6 — The frequencies of W→S, S→W and CpG methylation deamination process around the ends of CGI-genes in vertebrates. The frequencies S→W (thin lines) are calculated without the substitution in CpGs and compared to W→S frequencies (thick lines). The methylation deamination rates CpG→TpG (thick) and CpG→CpA (thin) are presented in the right panels. See Additional file 1 for further details. [file 1471-2148-10-187-S6.PNG]

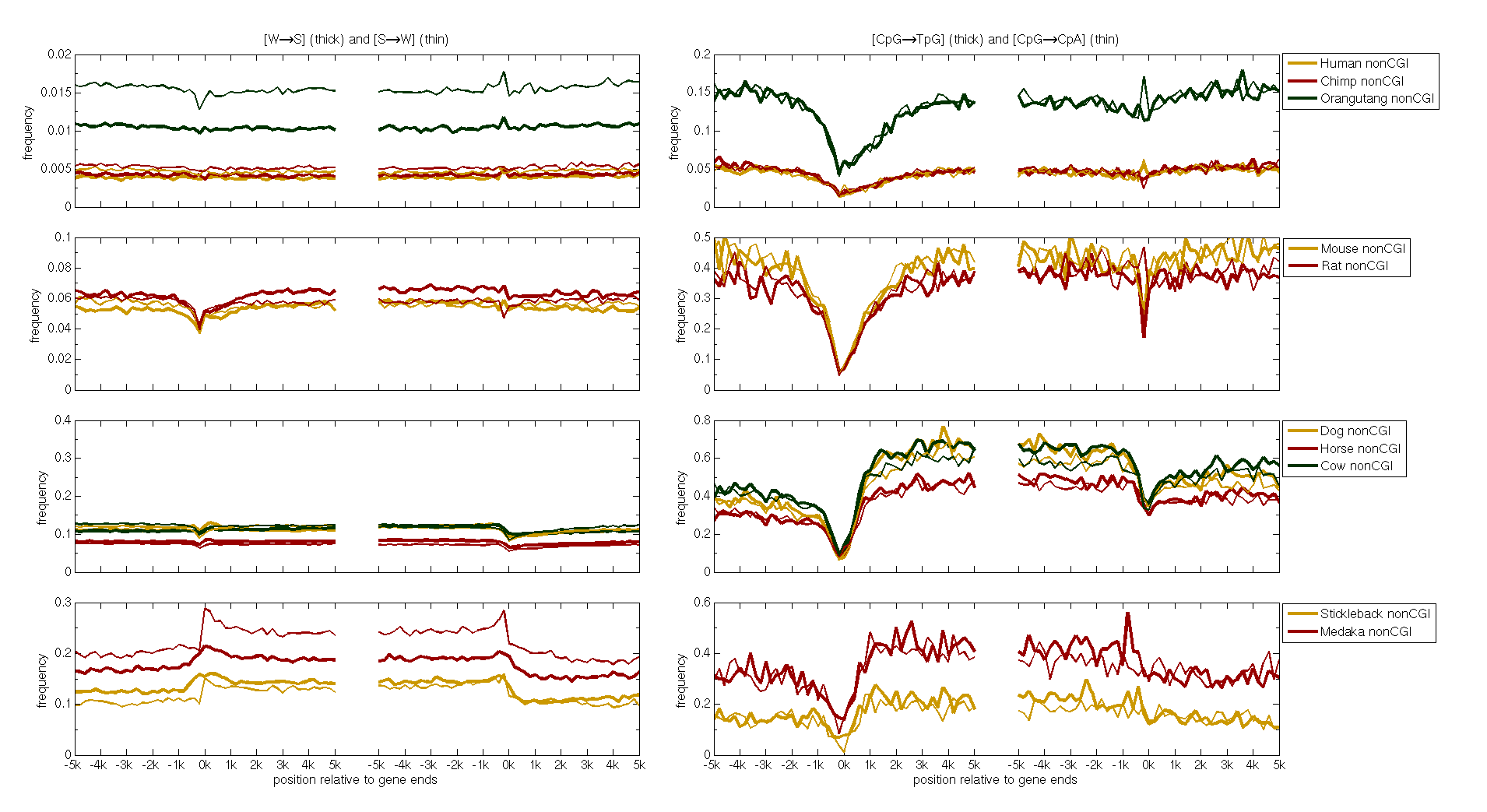

Supplement: Additional file 7 — Portable Network Graphics (PNG). The frequencies of W→S, S→W and CpG methylation deamination process around the ends of nonCGI-genes in vertebrates. The frequencies S→W (thin lines) are calculated without the substitution in CpGs and compared to W→S frequencies (thick lines). The methylation deamination rates CpG→TpG (thick) and CpG→CpA (thin) are presented in the right panels. See Additional file 1 for further details. [file 1471-2148-10-187-S7.PNG]

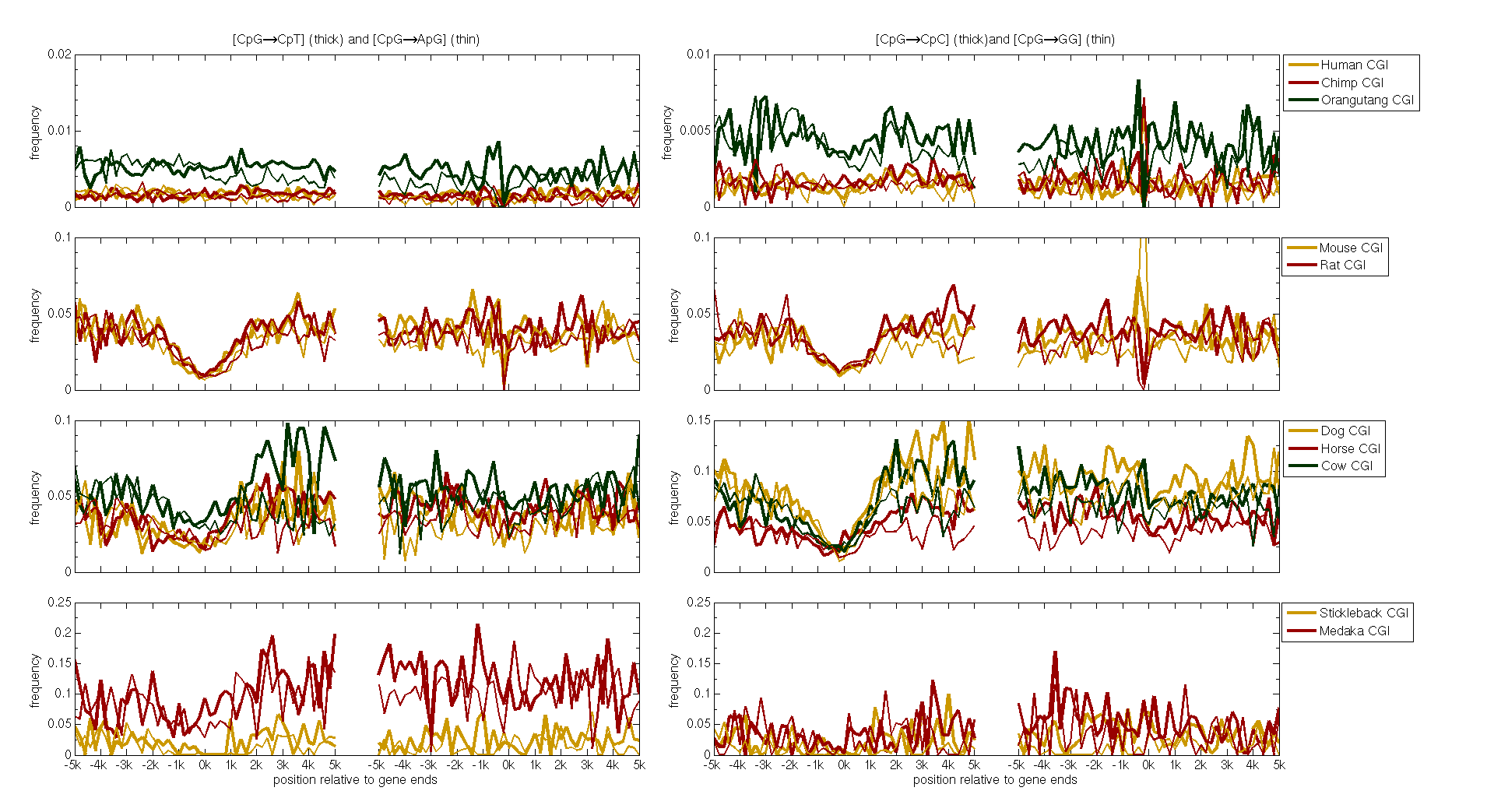

Supplement: Additional file 8 — Transversion rates that are associated with CpG methylation deamination process around the ends of CGI-genes in vertebrates. The methylation deamination rates CpG→CpT (thick) and CpG→ApG (thin) are presented in the left panels. The rates CpG→GpG (thick) and CpG→CpC (thin) are presented in the right panels. See Additional file 1 for further details. [file 1471-2148-10-187-S8.PNG]

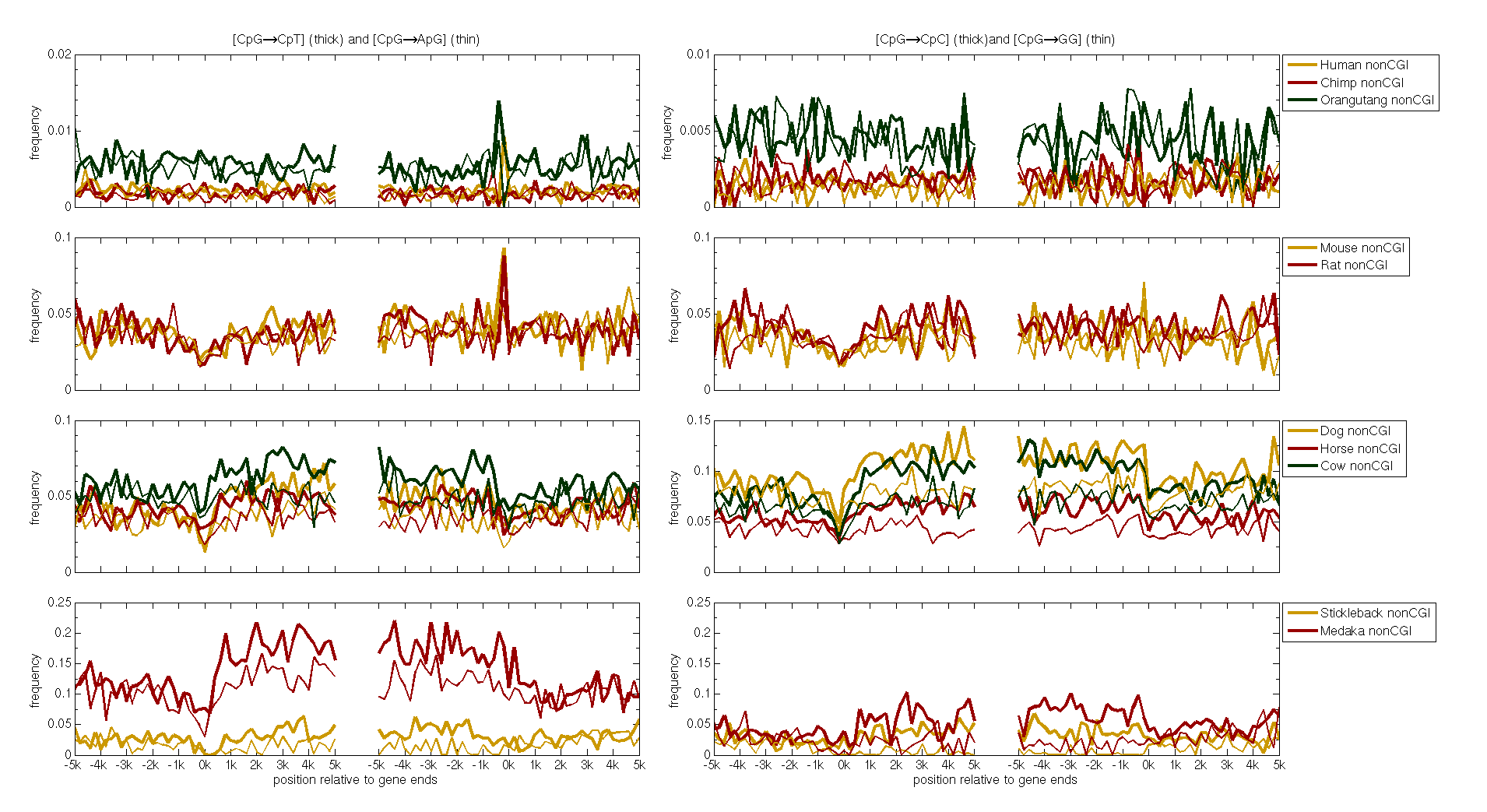

Supplement: Additional file 9 — Transversion rates that are associated with CpG methylation deamination process around the ends of nonCGI-genes in vertebrates. The methylation deamination rates CpG→CpT (thick) and CpG→ApG (thin) are presented in the left panels. The rates CpG→GpG (thick) and CpG→CpC (thin) are presented in the right panels. See Additional file 1 for further details. [file 1471-2148-10-187-S9.PNG]

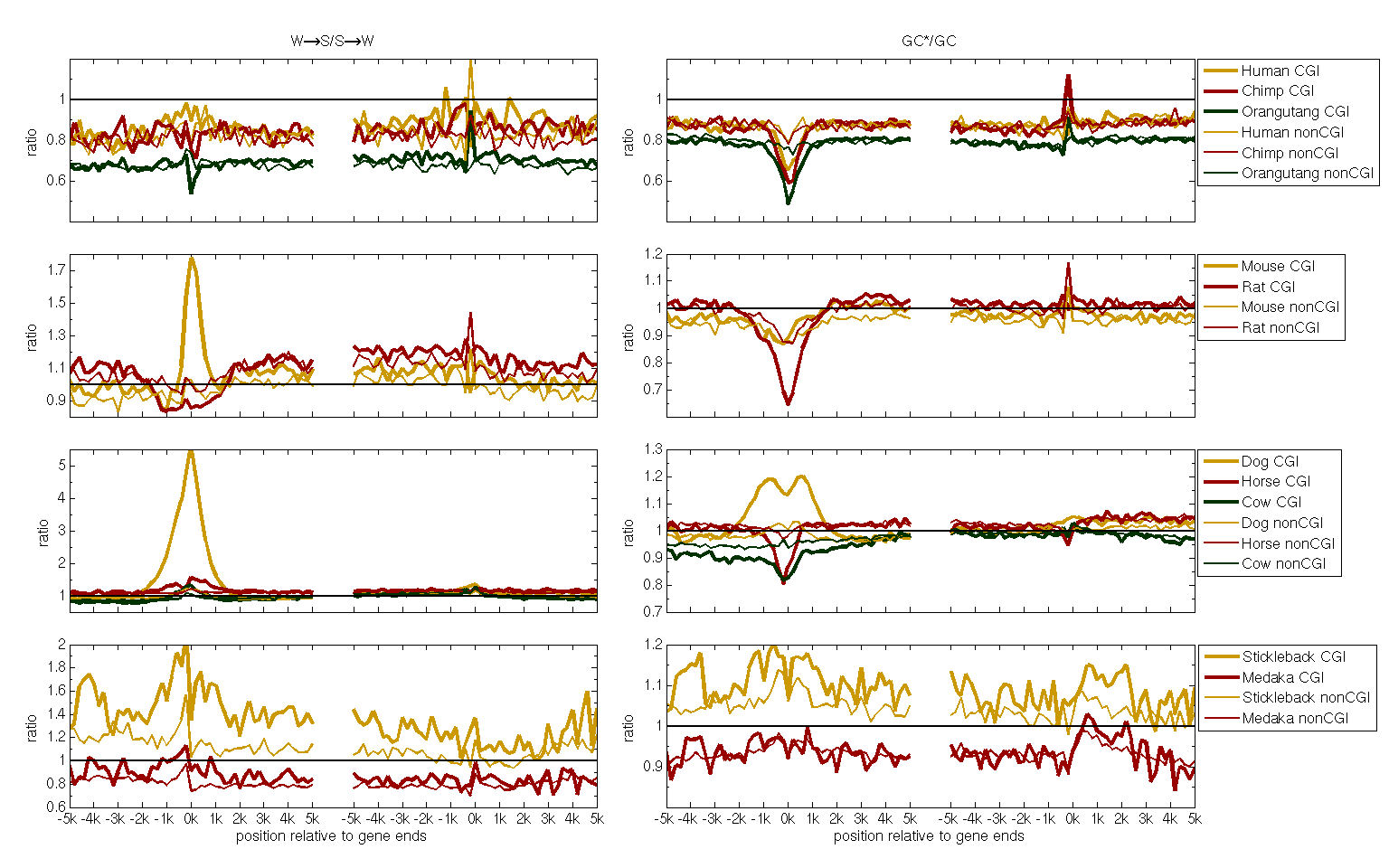

Supplement: Additional file 10 — Profiles of [W→S]/[S→W] and GC*/GC ratios across vertebrates. For CGI-genes the ratios are presented by thicker lines. See Additional file 1 for further details. [file 1471-2148-10-187-S10.png]

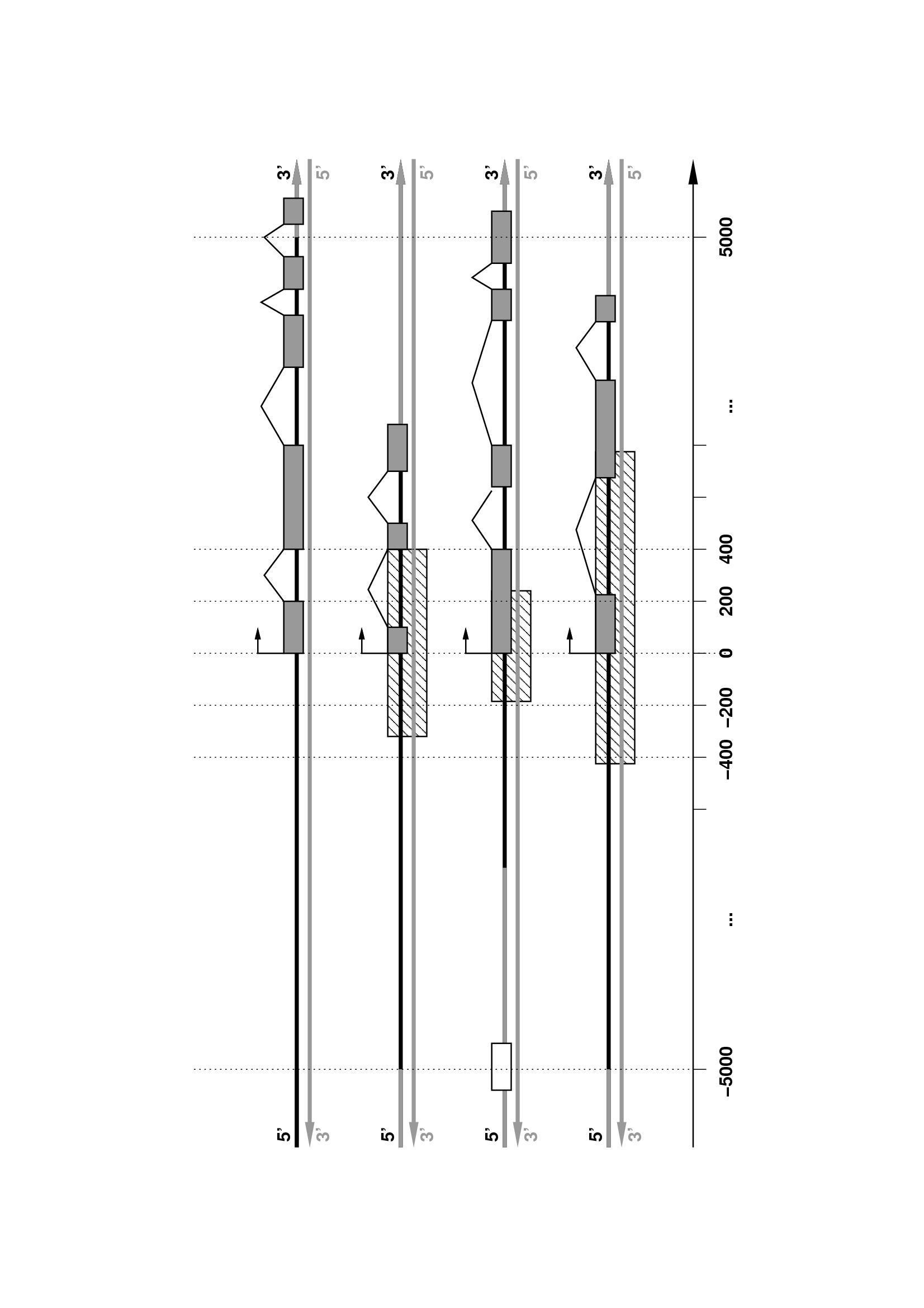

Supplement: Additional file 11 — Sketch of the analyzed regions around the 5' ends of two classes of genes. Genes starting within CGIs, denoted by striped boxes, and genes without CGI. CGIs are denoted by striped boxes. The template strand for transcription (also denoted by the coding strand) is the reference strand that is used for the substitution analysis and for defining the directionality 5'→3' relative to the 5' end (which denoted by 0) gene start (broken arrow). The substitution analysis was done in the 10000 bp long regions centered on the 5'end of gene (denoted by the two outmost vertical lines). This region of analysis was further truncated if the next upstream gene was closer than 10000 bp (white box) or the 3'end of the gene was closer than 5000 bp. Further all, exons were excluded (gray boxes). Bold black lines depict the finally analyzed sequences. The substitution frequencies are estimated relative to the 5'end of genes using a sliding window analysis. [file 1471-2148-10-187-S11.png]
